# Wing Variability in Some Andean Brown Lacewing Insects as an Adaptive Survival Strategy (Insecta, Neuropterida, Neuroptera: Hemerobiidae)

**DOI:** 10.3390/insects16040401

**Published:** 2025-04-11

**Authors:** Víctor J. Monserrat, Óscar Gavira

**Affiliations:** 1 Departamento de Biodiversidad, Ecología y Evolución, Facultad de Biología, José Antonio Novais, 12, Universidad Complutense, 28040 Madrid, Spain; 2 Departamento de Biología Animal, Facultad de Ciencias, Universidad de Málaga, Campus de Teatinos s/n, 29071 Málaga, Spain

**Keywords:** Insecta, Neuroptera, Hemerobiidae, adaptive color variability, camouflage, polymorphism, anti-predator defenses

## Abstract

The variability in shape and coloration patterns associated with strategies of crypsis increases the environmental entropy and makes it more difficult for a potential predator to learn a certain prey to locate. To demonstrate this concept, we composed images of the wings of two Hemerobiidae species (*Gayomyia falcata* and *Megalomus stangei*) on a leaf background and then optically analyzed them by calculating the entropy of the images (in color as well as grayscale). For comparison, we colored the wings of Hemerobiidae artificially, and the analysis was repeated with these non-cryptic wings. The results indicate that the artificially colored wings reduce the entropy of the image, facilitating the location of the specimen, while the natural wings increase the entropy, thus hiding the presence of the specimen. In this context, the more morphological and chromatic diversity that the wings show, the greater the increase in entropy.

## Simple Summary

The family of brown lacewings (Neuroptera, Hemerobiidae) shows many strategies of crypsis, including a brown general body; disruptive, dark variegated or maculated wings; falcate wings; and so on. On this basis, some species present a real marked variability in the shape and size of their wings, as well as a remarkably wide variation in wing coloration patterns, which do not seem to be affected by their geographical distribution, sex, or age. This variability makes it more difficult for a potential predator to learn a certain wing model to locate. In this contribution, we demonstrate the efficacy of such variability as an anti-predatory strategy used to maximize the survival and reproductive success of the species by avoiding or minimizing the risk of potential visual identification by predators.

## 1. Introduction

The survival capacity of species in the Animal Kingdom—and particularly insects—is increased through reducing the risk of being predated by other animals that hunt them by sight. Although many groups present bright and conspicuous color patterns—mostly related to territoriality, reproduction, or aposematism—the majority of insects generally rely on structural or color camouflage (i.e., crypsis), with the aim of becoming less conspicuous and imitating the form, external aspect, and/or general coloration of their surroundings, both physically and chemically (smell, touch, sound, etc.) but namely visually. These forms of mimicry and crypsis, especially among the insects, comprise a very generalized survival strategy [1,2,3,4,5,6,7,8,9].

The Superorder Neuropterida includes Megaloptera (c. 380 spp. extant), Raphidioptera (c. 250 spp. extant), and Neuroptera (c. 6000 spp. extant), the latter being the most diverse order, with about 19–21 extant families (according to the opinions of different authors), some of which are considered authentic living fossils (according to the criteria of Thenius [10]) that, after millions of years, have survived to the present day [11,12,13,14,15]. They have a cosmopolitan distribution (except for Antarctica) with elements that, in many cases, have very fragmented distributions and almost relict positions.

In the order Neuroptera (a small group, compared to other orders of holometabolous insects), an enormous diversity in both morphology and appearance can be observed, as well as disparate biology and ways of life of these insects, both in their imago as well as larval stages.

Aside from the general evolutionary adaptations of holometabolous insects, among Neuropterans we find a multitude of anatomical, morphological, physical, chemical, biological, physiological, and/or behavioral strategies and resources that facilitate their survival and procreation. In this contribution, we will focus on defense mechanisms of a visual nature, either passive (coloration and integumentary structures) or active (specific behaviors).

As for other representatives of the Animal Kingdom, one of the passive protection mechanisms that Natural Selection has provided to many insect species is the widespread aposematic, cryptic–mimetic, and/or disruptive colorations [1,2,3,6,7,16,17,18]. These adaptations have allowed for the success of these species and obviously occurred in ancient times; notably, they are also observed in the group that concerns us [19,20,21]. Within the different families, we find many examples of the presence of disruptive, cryptic, body position, or mimicry strategies in body and wing coloration patterns, which not only help to break up the outline of the individual (thus hindering its identification as prey), but specifically simulate the surrounding environment in which these species live or serve to imitate other species. This is in addition to some other special and peculiar patterns and behaviors (as observed in Ascalaphidae, Myrmeleontidae, Nemopteridae, Mantispidae, Berothidae, Psychopsidae, and so on), involving certain unusual resting positions of the body (or part of the body) that make them less vulnerable to predation; for example, by camouflaging with the leaves, branches, or stems where they rest.

The presence of eyespots on the wings (usually called ocellus) imitating a bigger or different animal, death-feigning behavior (thanatosis), the secretion of repellents or paralyzing substances, or some specialized flight patterns in response to the calls of echolocating flying bats are well-known adaptive responses to survival which are relatively common in different families. Additionally, the behavior of the larvae of some Chrysopidae that cover themselves with external elements to go unnoticed is well known, and other predatory families, such as Mantispidae or some larvae in Myrmeleontidae, may assume the same color patterns as the background on which they hunt and even, through Batesian mimicry, imitate the structures and coloration of other insects—such as social wasps, bees, or sand wasps—to protect themselves [13,22,23,24,25,26,27,28,29,30,31].

**Figure 1:**
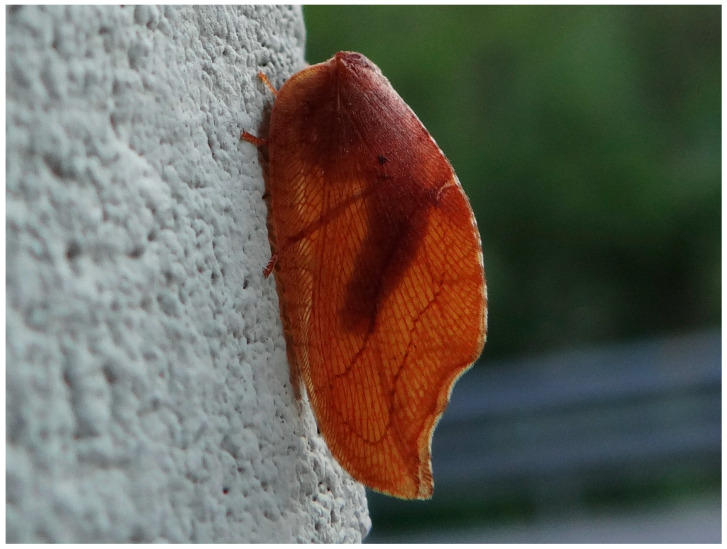
*Drepanepteryx phalaenoides* (Linnaeus, 1758) (photo by M. Nadal).

**Figure 2:**
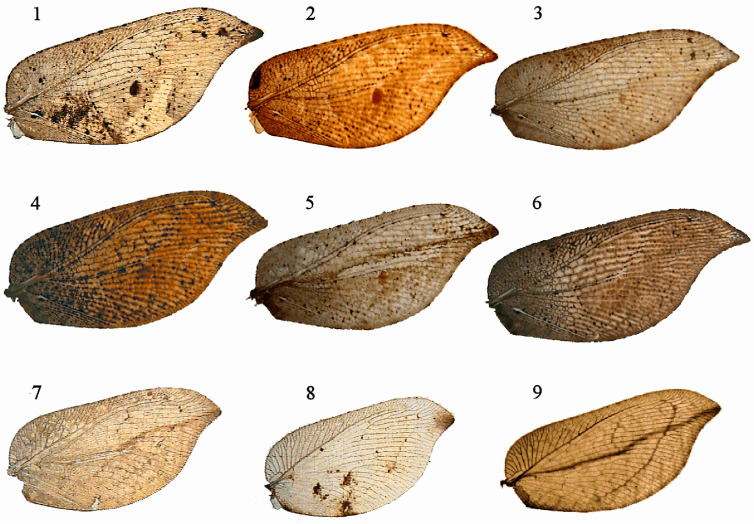
Images 1–6: Forewings of *Gayomyia falcata* (Blanchard in Gay, 1851) specimens from 1: Chile, Lemuy, San Agustín (GAY-1); 2: Chile, Chiloé, Dalcahué, (GAY-2); 3: Argentina, Neuquén, Villa de la Angostura (GAY-3); 4: Chile, Última Esperanza, Refugio del Lago Toro (GAY-4); 5: Chile, Chiloé, Dalcahué (GAY-5); and 6: Argentina, Neuquén, Villa de la Angostura (GAY-6). Images 7–9: Forewings of *Megalomus stangei* González Olazo, 1981 specimens from 7: Chile, Última Esperanza, Refugio del Lago Toro (MEG-1); 8: Chile, Chiloé, Hullinco (MEG-2); and 9: Chile, Osorno, Lago Rupanco (MEG-3). Photo by E. Ruiz.

One of the Neuropteran families is the brown lacewing or Hemerobiidae, with c. 600 current species and a cosmopolitan distribution (except Antarctica, some very isolated oceanic islands, and deserts without vegetation). Most of their species present a brown (exceptionally green) general coloration pattern of the body and wings, sometimes with disruptive, darker variegated or maculated wing membranes. With these color patterns, they try to rest camouflaged on the vegetation where they live by resembling the general color pattern of their background [30,32,33]. As another visual defensive mechanism, as observed in some genera such as *Micromus*, *Gayomyia*, *Drepanepteryx*, *Megalomina*, *Drepanacra*, or *Megalomus*, some species have falcate wings [34,35,36,37] that simulate a small piece of dry and dead leaf (even imitating leaf venation and a possible incurring leaf attack by phytophages or fungi) as another camouflage strategy (Figure 1 and Figure 2); similar strategies occur in many other insects (Orthoptera: Acrididae; Dictyoptera: Mantidae; Coleoptera: Elatheridae, Buprestidae, Curculionidae, Chrysomelidae, and Coccinellidae; Lepidoptera: Nymphalidae; and so on).

As in some other Neuropteran families—such as Dilaridae, Chrysopidae, Berothidae, Osmylidae, Coniopterygidae, or Sisyridae, whose adults also live on vegetation—most of the species of Hemerobiidae have another protection and survival strategy: the defensive behavior of death-feigning (a specialized type of thanatosis), in which they fall down from the plant to the substrate, closing their wings to protect their head, antennae, legs, and body. In this way, they simulate death, remaining motionless in this position on the carpet of leaf litter for as long as the potential danger remains [12,30,38].

The specially sclerotized forewings in some of the Hemerobiidae species of these genera (also in other genera, such as *Hemerobius*, *Conchopterella*, *Notiobiella,* or *Psectra*) could increase the efficacy of such defensive behavior [12,13,34,39,40,41,42,43,44,45], and the combination of brown-falcate cryptic wings and this thanatosis behavior—as a multiple defensive mechanism—is highly effective when escaping from their potential predators, and is considered an adaptive strategy to maximize survival and reproductive success.

It is also especially curious and unusual that, particularly in most of the species of these related genera with falcate wings (*Gayomyia*, *Drepanepteryx*, *Megalomina*, *Drepanacra, Conchopterella*, or *Megalomus*), there is marked variability in the shape and size of their wings and, in particular, a remarkably wide variation in wing coloration patterns [35,37,46,47] (Figure 2). This phenomenon has also been observed in some species of other genera, such as *Carobius* or *Micromus* [34,35,45,48], even between specimens of the same population, and it does not seem to be affected by the geographical distribution, sex, or age of the specimens (Figure 2), seeming to only be the consequence of wide phenotypic plasticity and genetic variability. Such unusually rich variation in the coloration pattern of these brown lacewings has been recorded as a very interesting fact that merits further biological attention [35].

Finally, it is also interesting that some of these related falcate and variable wing species (quoted above) are mostly linked to relict Gondwanian deciduous forests in the southern continents (mostly to *Nothofagus* Blume, 1850: Nothofagaceae), for example, in the south-west of South America and some other wet deciduous forests in the south-east of Australia, Tasmania, New Zealand, New Guinea, and New Caledonia, in which a permanent dead and dry brown leaf litter is observed.

In this contribution, we demonstrate the efficacy of such variability as an efficacious strategy to maximize the survival and reproductive success of species by avoiding or minimizing the risk of potential visual identification by predators.

## 2. Materials and Methods

Among the recorded genera whose species show an unusually wide variability in wing shape and color pattern, and which also present falcate cryptic wings, we selected two South American species: *Gayomyia falcata* (Blanchard in Gay, 1851) and *Megalomus stangei* González Olazo, 1981 (assigned to genus *Conchopterella* Handschin, 1955 according to Oswald [36]) (Figure 2). Both are widely sympatric, mostly confined to areas of Andean influence from Bolivia, Chile, and Argentina, and are strongly linked to *Nothofagus antarctica* (G. Forster) Oerst. forests [37,47,49].

**Figure 3:**
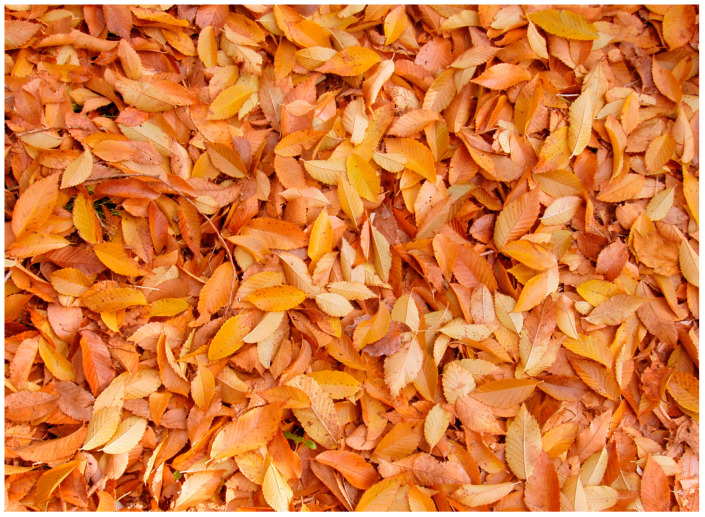
Image of dead leaf litter from *Ulmus minor*.

**Figure 4:**
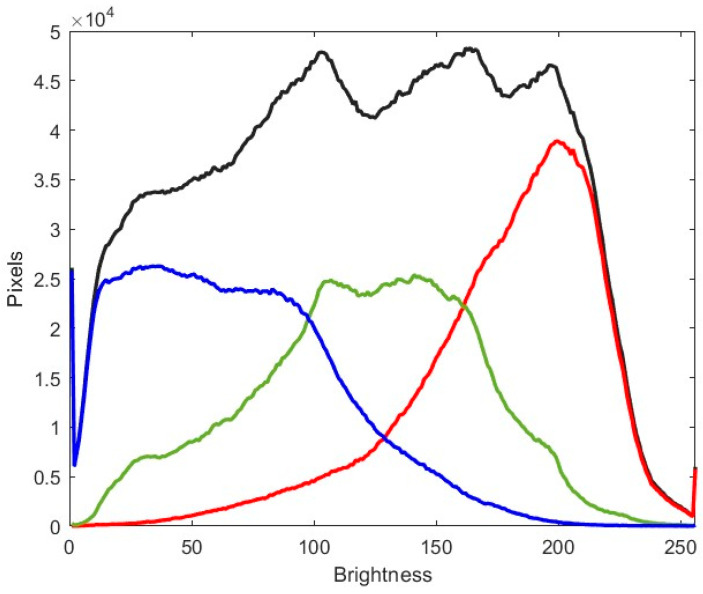
Histograms of the leaf background (Figure 3). The histogram of the grayscale images in black. The histogram of each color channel in the corresponding color.

In order to demonstrate whether such variability in wing shape and coloration is optically effective to avoid or minimize the risk of being detected by any potential visual predator (including birds, frogs, lizards, or other insects, being able or not to see in color)—and, therefore, whether it may be an effective adaptive survival strategy—we obtained an initial colored photograph simulating deciduous *Nothofagus* dead brown leaf litter (using dead leaf litter of the European Ulmaceae, *Ulmus minor* Miller, which has similar leaves; Figure 3). The histogram of this dead leaf litter image is shown in Figure 4.

To this initial image, we added the forewing images (the visible aspect in a thanatosis situation) of our specimens. The specimens came from V. J. Monserrat’s collection, which was deposited in the Complutense University of Madrid, Biological Science Faculty. Wing images were taken using an Olympus^®^ SZX7 stereomicroscope with an integrated camera (Olympus S-C-30) under the same conditions (brightness, contrast, exposure, etc.) and processed using the Olympus analySIS getIT software. We used 6 types of wings from *Gayomyia falcata* and 3 types from *Megalomus stangei* (Figure 2).

**Figure 5:**
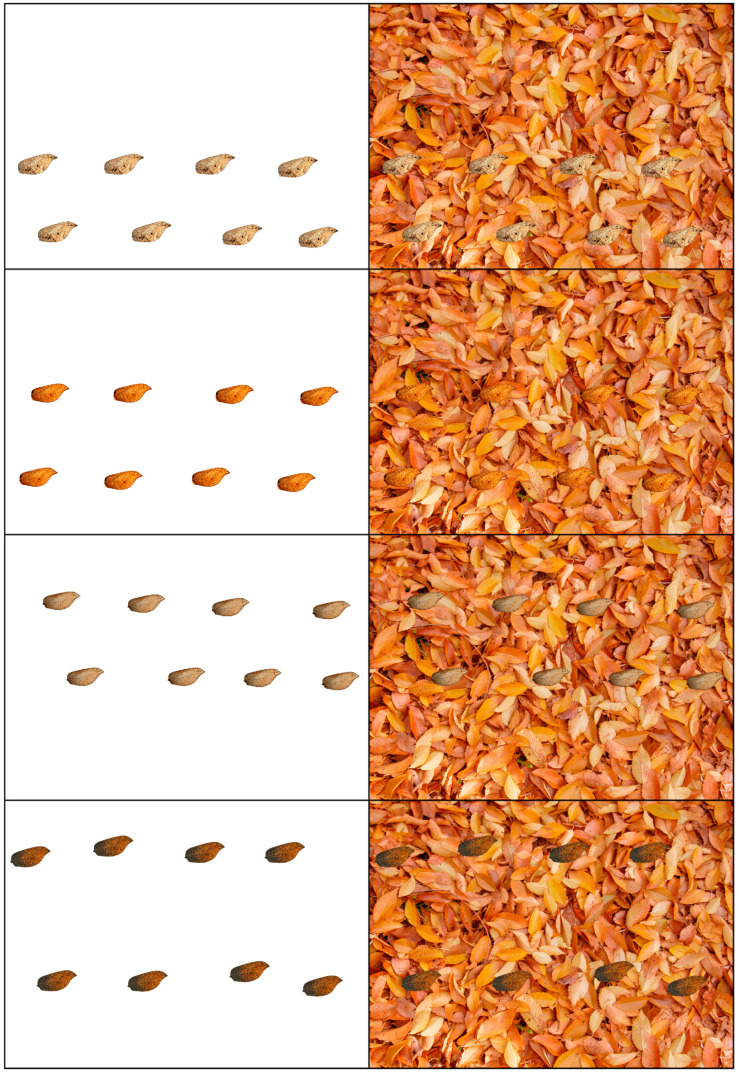
Four selected forewing specimens of *Gayomyia falcata* (from GAY-1 to GAY-4), each with eight wings, superimposed over the white background and the image of dead leaf litter from *Ulmus minor*.

**Figure 6:**
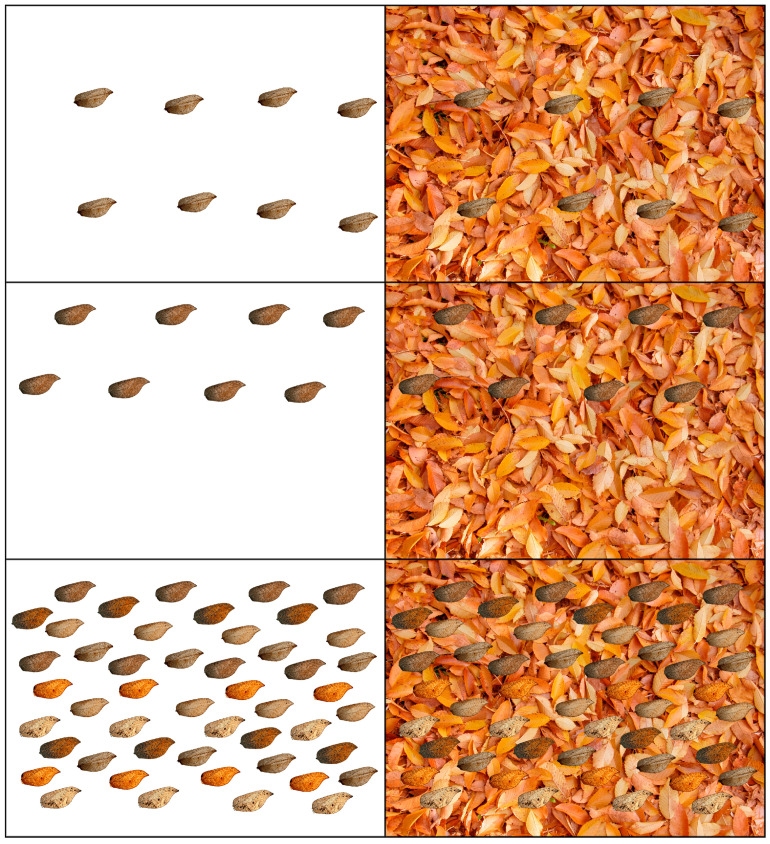
Two selected forewing specimens of *Gayomyia falcata* (GAY-5 and GAY-6), each with eight wings, and all types of forewings, each type with eight wings, superimposed over the white background and the image of dead leaf litter from *Ulmus minor*.

**Figure 7:**
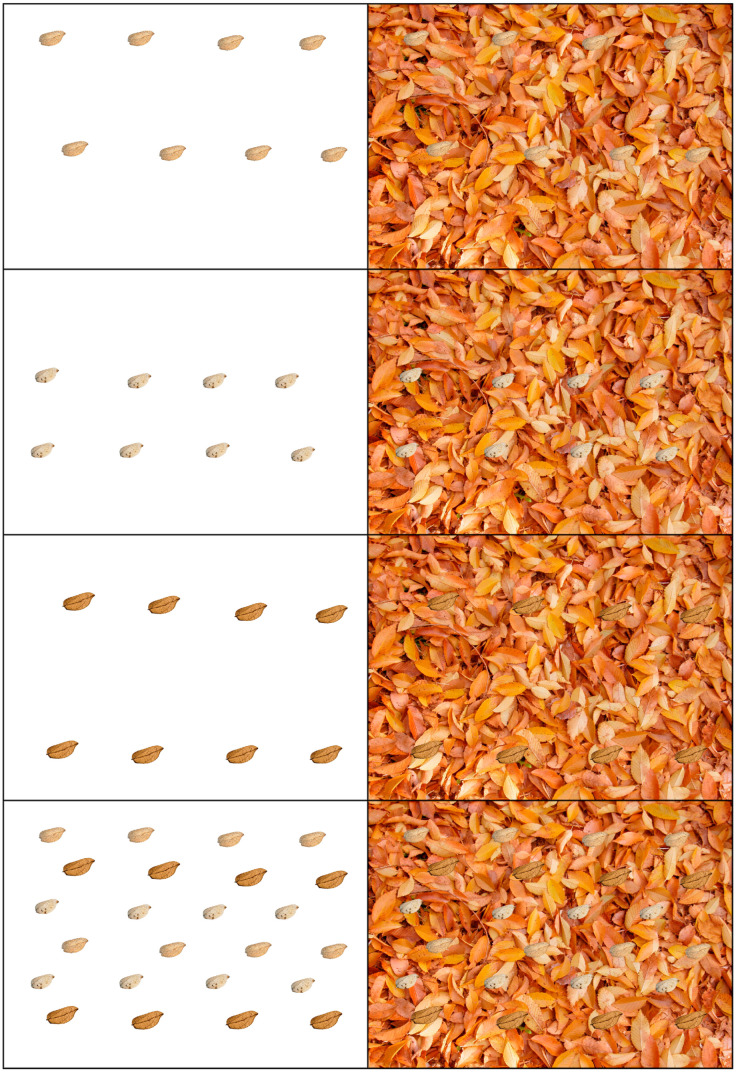
Three selected forewing specimens of *Megalomus stangei* (from MEG-1 to MEG-3), each with eight wings, and all types of forewings, each type with eight wings, superimposed over the white background and the image of dead leaf litter from *Ulmus minor*.

Images for each type of wing were composed with 8 copies of each wing, and a final image for each species that integrated all types of wings, also with 8 copies of each type; for *Gayomyia falcata*, this resulted in a total of 48 wing silhouettes, while for *Megalomus stangei*, there were 24 wing silhouettes. These compositions were optically analyzed and compared to the wings in the absence of the leaf background (white background). In order to avoid effects derived from a change in the position of the copies of the wings, the same location in the image was maintained, such that the result exclusively reflected the changes in the environment (Figure 5, Figure 6 and Figure 7).

**Figure 8:**
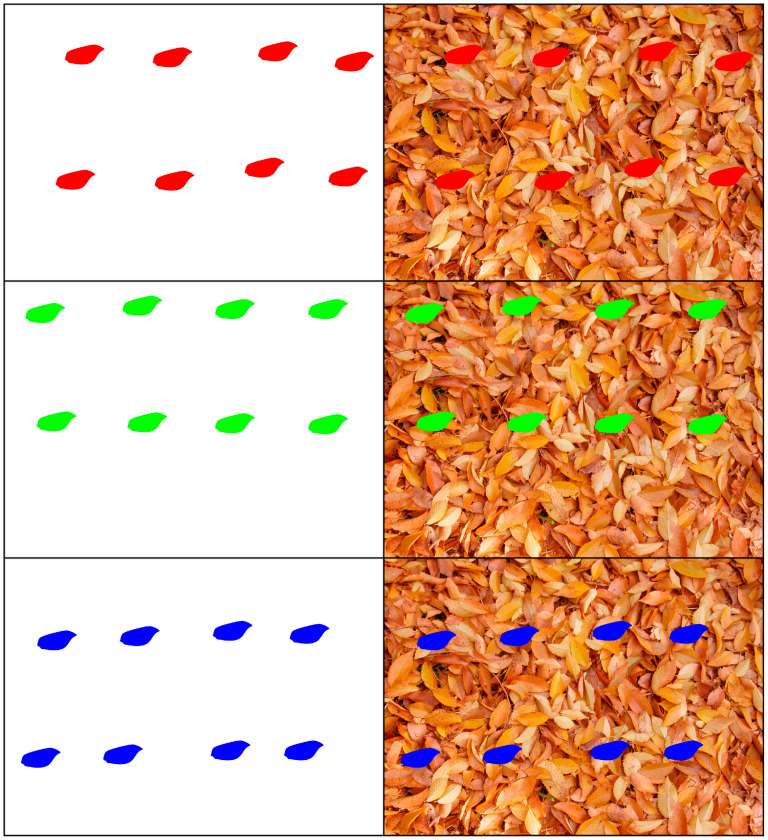
Wings artificially colored with the primary colors (red, green, and blue), each color with eight wings, superimposed over the white background and the image of dead leaf litter from *Ulmus minor*.

**Figure 9:**
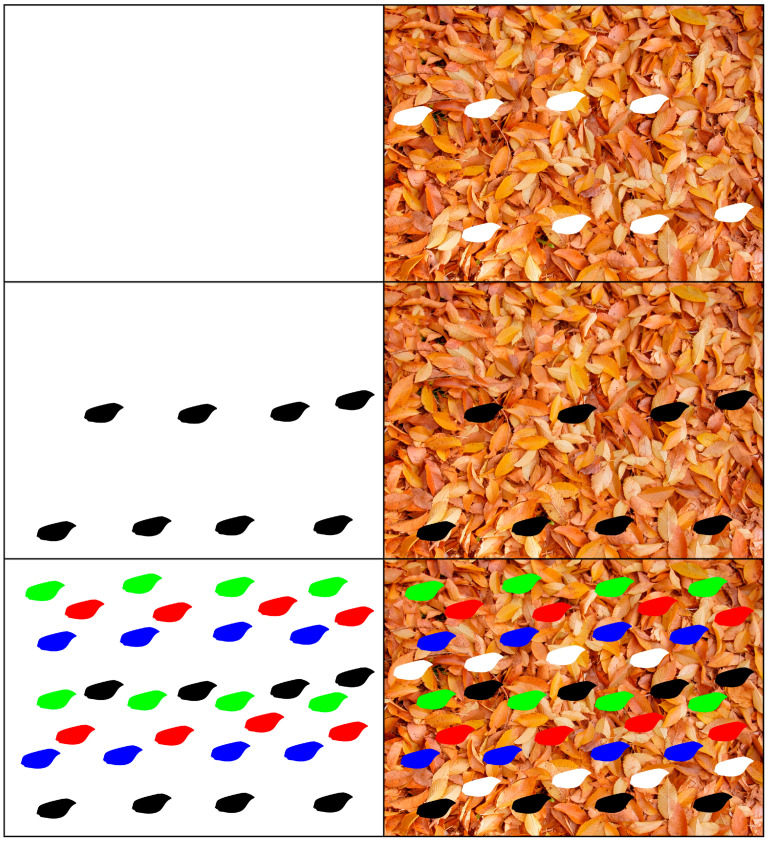
Wings artificially colored with black and white, each with eight wings, and all the colored forewings, each color with eight wings, superimposed over the white background and the image of dead leaf litter from *Ulmus minor*.

Furthermore, to analyze the effect of a hypothetical non-cryptic wing, this same procedure was repeated using wings that were artificially colored with the primary colors (red, green, and blue), in addition to black and white (Figure 8 and Figure 9).

In order to demonstrate whether such variability is also optically effective to avoid or minimize the risk of being detected by any potential visual predator that is not able to see in color, we also repeated the same figure compositions while eliminating the color, resulting in black, gray, and white images.

The MATLAB^®^ R2022b program was used to calculate the entropy of the images. Entropy is defined as a statistical measure of randomness, which is defined according to the formula -sum(p·log2(p)), where p denotes the normalized histogram counts (adapted from Shannon [50,51]). To calculate the entropy of grayscale images, the formula was applied to the image histogram, which, by default, is represented in grayscale. To calculate the entropy of color images, the entropy of each primary color (color channel) was calculated from the histograms for each color (red, green, and blue). Thus, we define the entropy of a color image as the sum of the entropies of the three primary colors. Therefore, grayscale and monochrome images have a maximum entropy of 8 bits, while color images have a maximum entropy of 24 bits. The results are expressed both in bits and as a percentage of the maximum entropy value in each case.

After the results of the image entropy analysis, we first tested the natural and artificially colored wings on the white background to compare the value between them using Student’s *t*-test, with *p* < 0.05. We also compared the entropy of the total color and the grayscale images to the value of the leaf background with Student’s *t*-test (*p* < 0.05). The statistical analyses were conducted using the IBM SPSS Statistics version 20 software. In both analyses, the mixed wings were excluded due to their greater number of wings.

## 3. Results

The obtained results are summarized in Table 1 and Table 2. The entropy of the white background was null, but the image of the leaf background indicated a high-entropy scenario, being close to the maximum value (91.6% for the color image and 98.2% for the grayscale image; Table 1 and Table 2). The color histograms of the leaf background indicated that the different channels were spread throughout the spectrum; in this sense, the red channel showed lighter tones and lower entropy, the blue channel showed darker tones and intermediate entropy, and the green channel showed the most balanced tones and the highest entropy (Figure 4).

The measurements made on the white background showed low values for all types of wings (Table 1). The color values were always lower than the gray values, and the result of mixing the different types of wings was always higher than each type separately (2–5 times higher). The entropy of the artificially colored wings showed values similar to those obtained in *M. stangei*, although slightly lower (except for the white color, which was null). However, there were no significant differences between them. The entropy of *G. falcata* showed the highest values, both for each type of wing separately (7–8%) and for the image that integrates all types (35.7% for the color image and 36.4% for the grayscale image). These differences between *G. falcata* and the others are statistically significant.

On the background of leaves, the colored wings reduced the entropy of the background in all cases, and the reduction was higher in the images that integrated all the colors, with a reduction of 5.1% in the color image and a 6.2% reduction in the gray image. The smallest reduction was 0.36% for the blue wings in the color image, and 0.23% for the green wings in the gray image.

On the contrary, in all cases, the cryptic wings increased the entropy of the background in the color image, with the highest increase observed for the mixture of wings (1.4% for *G. falcata* and 0.52% for *M. stangei*) (Table 2). In the gray image, the wings increased the entropy of the background except in four cases in which it was slightly reduced (MEG-3, GAY-3, GAY-5, and GAY-6), where the largest decrease was 0.03% (GAY-3), which is much lower than the minimum reduction produced by the colored wings in the gray image (0.23% for the green wings); therefore, in these cases, the entropy of the background remained high. In the two species, the mixture of wings produced the highest increase in background entropy in the gray image (0.11% for *G. falcata* and 0.06% for *M. stangei*). The entropy increases in the gray images were less than those in the color images, as the background image in gray already had a very high entropy, which was close to the maximum (98.2%).

Student’s *t*-test indicated that the increase in the image entropy due to the wings of *G. falcata* was statistically significant in the total color images, but not in the grayscale images. Meanwhile, the results for *M. stangei* were not statistically significant. In the colored wings, the decrease was statistically significant in both total color and grayscale images.

## 4. Discussion

In this study, the effects of cryptic and artificially colored non-cryptic wings were compared in both a scenario without entropy (white background) and one with high entropy (background of leaves). The white background allowed us to compare the entropy associated with both species due only to the coloration of the wings, and not to the environment, showing different values for the two species of Neuroptera. On the contrary, the non-cryptic wings, despite having been colored artificially, showed similar values to those of *M. stangei*. However, on the background of leaves, it was found that the wings colored with bright colors reduced the entropy of the image, facilitating access to information (i.e., the presence of the specimen), while the cryptic wings increased the entropy of the whole (thus hiding this information) or maintained it at a similar level; in particular, the more morphological and chromatic diversity that the wings show, the greater the increase in entropy. The wings of *M. stangei* did not significantly increase the entropy of the leaf background but maintained the existing level. On the contrary, the colored wings reduced the entropy of the leaf background, despite presenting similar values to the *M. stangei* wings on the white background. In grayscale, the effects of the natural wings on the leaf background were not significant due to the high entropy of this scenario without color; however, they did not reduce it, as was observed for the artificially colored wings. A new design would be needed to test the effect of the wing variability on the background in grayscale.

While this study focused on an analysis of the color variability, the wings of these species are polymorphic, and the effect of the morphological variability was not tested. Nevertheless, the considered wings integrated both color variability and morphological variability, although the latter was smaller. Further research is necessary to separately evaluate the effect of morphological variability on environmental entropy. The study in grayscale is insufficient because the color pattern would be different and recognizable without color, but it would provide an approximation.

As shown in the two selected Hemerobiidae species (Figure 2), some other insects that also rely on camouflage for survival often exhibit extreme individual variations, showing a great phenotypic variability of a single genotype to express different phenotypes in the same or different environments. Some known hypotheses and examples have been described in different aquatic and terrestrial organisms, but particularly among the insects, with some of the most-studied and best-known examples including the East Asian tropical butterfly *Melanitis leda* (Linnaeus, 1758) and the South Eastern African tropical butterfly *Bicyclus anynana* (Butler, 1879) (Lepidoptera: Nymphalidae, Satyrinae), which present adaptive phenotypic plasticity, not only showing seasonal polyphenism with distinct dry/wet season color patterns, but also a wide variability of color and shape during the dry season, when the butterflies rely on survival through crypsis on a resting background of dead brown leaves, making it difficult to find two individuals with exactly the same color pattern [52,53,54,55,56,57,58]. Among the Neuroptera, changes in general color at the time of initiating hibernation are also quite widespread in some genera of Chrysopidae, going from the spring–summer green to the autumn–winter brown [59,60,61].

This extreme polymorphism is also observed in some aquatic or terrestrial invertebrate inhabitants on the sea rock coast, dead leaf litter, or the soil surface, in a very wide range of habitats from deciduous woodland to sand dunes. The European snails of the genus *Cepaea* (Pulmonata: Helicidae) are a classical example that have been fully studied and recorded by Arndt [62], Cain [63], and Cook [64], among others.

A similar question arises for the two considered South American brown lacewing species (Figure 2), where a real seasonal polyphenism can hardly be applied due to the short life of these insects as imagoes and the relatively short placid climatic and environmental conditions when these insects can develop their imago activity [37,49]. Regarding their available lifetime activity as imagoes (all available data for collected *Gayomyia falcata* specimens show an annual amplitude from October to April, mostly concentrated in December, in localities between 80 and 1400 m within a 40º latitudinal amplitude, while, for *Megalomus stangei*, these range from September to June, mostly concentrated in February, in localities between 100 and 1800 m within a 15º latitudinal amplitude), they show an evidently wide variability in color, making it difficult to find two individuals with exactly the same color pattern (Figure 2). Furthermore, the age, geographical location, and/or sex of the specimens do not seem to have a relevant effect on the species of these two genera (the possible sexual dimorphism given by Wise [65] for the Australian *Drepanacra binocula* (Newman, 1838) seems not to be followed by the selected South American genera).

Other species of Neuroptera show polymorphism, as in *Climaciella brunnea* (Say in Keating, 1824) (Mantispidae), whose different color morphs have been described [25]; however, in this case imitating—via Batesian mimicry—different colors or aspects present in species of polistine wasps (*Polistes*, *Synoeca*). As in the butterflies or snails recorded, in the considered brown lacewing species, and also probably among the other already recorded genera (*Drepanepteryx*, *Megalomina, Conchopterella* or *Drepanacra*), the wide variation in the shape and color of the wings across individuals is considered to be produced by a high genetic variation as an evolutionary response involving apostatic selection, which has been suggested to make it more difficult for potential predators browsing in the leaf carpet to visually form a specific prey image for a particular form of dead leaf pattern corresponding to the color and shape patterns of the prey. As predation by lizards or birds may select and enhance such polymorphism, it may act apostatically to favor distinct morphs [3,7,18,64].

The variable and cryptic wings of *Gayomyia falcata* have been particularly recorded by Monserrat [37], and some curious defensive behaviors in association with the leaves of *Ribes magellanicum* Poiret, 1812 (Grossulariaceae) have also been recorded by Faúndez [33] in Magallanes (Chile) (as in other species [66]), as well as with respect to the leaves of *Nothofagus*. These morphological and behavioral responses to deciduous leaf litter are present in these insects and are reflected in the same aspect of the wings (both in shape and color). A possible Müllerian mimicry, which is beneficial for both studied species (both inhabitants of similar environments), can be suggested as another new survival strategy, and even a possible Batesian mimicry (as described by Morton [32] between the similar Palaearctic brown lacewing *Drepanepteryx phalaenoides* Linnaeus, 1758 (Figure 1) and the moth *Drepana lacertinaria* Linnaeus, 1758 Lepidoptera: Drepanidae) with other insect inhabitants in the same Andean environments presenting a similar aspect and behavior could be suspected.

Now that this hypothesis has been demonstrated as a physical–optical reality, although the defensive mechanism in insects must be taken as a complex of several interactive strategies (chemicals, sound, color, aspect, behavior, etc.), and the crypsis in insects is not made for our eyes, it can now be demonstrated that the variability in wing shape and color in the selected insects—in addition to other visual tactics, such as falcate wings, cryptic and possible Batesian–Müllerian mimicry, thanatosis, and so on—can be considered as an effective and adaptive multi-interactive strategy to maximize their survival and reproductive success.

## Figures and Tables

**Table 1:** Entropy of the images (in color and grayscale) with a white background and percentage of the maximum value (24 bits for color images and 8 bits for grayscale images and for each color channel).

White Background	Red	Green	Blue	Color	Grayscale
Background	0.0000 (0.00%)	0.0000 (0.00%)	0.0000 (0.00%)	0.0000 (0.00%)	0.0000 (0.00%)
					
**Colored wings**					
Red	0.2512 (3.14%)	0.2977 (3.72%)	0.3029 (3.79%)	0.8517 (3.55%)	0.3170 (3.96%)
Green	0.2987 (3.73%)	0.0345 (0.43%)	0.3106 (3.88%)	0.6438 (2.68%)	0.2486 (3.11%)
Blue	0.2992 (3.74%)	0.2948 (3.69%)	0.2630 (3.29%)	0.8570 (3.57%)	0.3177 (3.97%)
White	0.0000 (0.00%)	0.0000 (0.00%)	0.0000 (0.00%)	0.0000 (0.00%)	0.0000 (0.00%)
Black	0.2838 (3.55%)	0.2838 (3.55%)	0.2838 (3.55%)	0.8515 (3.55%)	0.2838 (3.55%)
Colored wing mix	0.8980 (11.22%)	0.6957 (8.70%)	1.0029 (12.54%)	2.5965 (10.82%)	0.8875 (11.09%)
					
**Gayomyia falcata**					
GAY-1	0.6075 (7.59%)	0.6231 (7.79%)	0.6227 (7.78%)	1.8534 (7.72%)	0.6262 (7.83%)
GAY-2	0.5777 (7.22%)	0.5812 (7.27%)	0.5493 (6.87%)	1.7083 (7.12%)	0.5952 (7.44%)
GAY-3	0.5515 (6.89%)	0.5508 (6.89%)	0.5498 (6.87%)	1.6522 (6.88%)	0.5607 (7.01%)
GAY-4	0.6515 (8.14%)	0.6234 (7.79%)	0.5928 (7.41%)	1.8677 (7.78%)	0.6422 (8.03%)
GAY-5	0.5594 (6.99%)	0.5589 (6.99%)	0.5590 (6.99%)	1.6772 (6.99%)	0.5652 (7.06%)
GAY-6	0.6125 (7.66%)	0.6011 (7.51%)	0.5942 (7.43%)	1.8079 (7.53%)	0.6112 (7.64%)
GAY-mix	2.8884 (36.10%)	2.8638 (35.80%)	2.8182 (35.23%)	8.5704 (35.71%)	2.9101 (36.38%)
					
**Megalomus stangei**					
MEG-1	0.3469 (4.34%)	0.3508 (4.38%)	0.3544 (4.43%)	1.0521 (4.38%)	0.3581 (4.48%)
MEG-2	0.3017 (3.77%)	0.3065 (3.83%)	0.3118 (3.90%)	0.9199 (3.83%)	0.3099 (3.87%)
MEG-3	0.4648 (5.81%)	0.4623 (5.78%)	0.4610 (5.76%)	1.3881 (5.78%)	0.4781 (5.98%)
MEG-mix	0.9936 (12.42%)	1.0119 (12.65%)	1.0338 (12.92%)	3.0393 (12.66%)	1.0298 (12.87%)

**Table 2:** Entropy of the images (in color and grayscale) with a leaf background and percentage of the maximum value (24 bits for color images and 8 bits for grayscale images and for each color channel).

Leaf Background	Red	Green	Blue	Color	Grayscale
Background	7.1780 (89.72%)	7.5146 (93.93%)	7.2896 (91.12%)	21.9822 (91.59%)	7.8523 (98.15%)
					
**Colored wings**					
Red	7.1458 (89.32%)	7.4737 (93.42%)	7.2214 (90.27%)	21.8409 (91.00%)	7.8176 (97.72%)
Green	7.1514 (89.39%)	7.4722 (93.40%)	7.2440 (90.55%)	21.8677 (91.12%)	7.8343 (97.93%)
Blue	7.1617 (89.52%)	7.4715 (93.39%)	7.2620 (90.78%)	21.8952 (91.23%)	7.8179 (97.72%)
White	7.1404 (89.26%)	7.4749 (93.44%)	7.2663 (90.83%)	21.8816 (91.17%)	7.7934 (97.42%)
Black	7.1467 (89.33%)	7.4651 (93.31%)	7.2188 (90.24%)	21.8306 (90.96%)	7.7777 (97.22%)
Colored wing mix	6.8097 (85.12%)	7.0149 (87.69%)	6.9454 (86.82%)	20.7699 (86.54%)	7.3597 (92.00%)
					
**Gayomyia falcata**					
GAY-1	7.2185 (90.23%)	7.5451 (94.31%)	7.3188 (91.48%)	22.0824 (92.01%)	7.8597 (98.25%)
GAY-2	7.2001 (90.00%)	7.5258 (94.07%)	7.2904 (91.13%)	22.0163 (91.73%)	7.8623 (98.28%)
GAY-3	7.2057 (90.07%)	7.5160 (93.95%)	7.2996 (91.24%)	22.0213 (91.76%)	7.8498 (98.12%)
GAY-4	7.2742 (90.93%)	7.5299 (94.12%)	7.2690 (90.86%)	22.0730 (91.97%)	7.8539 (98.17%)
GAY-5	7.2313 (90.39%)	7.5174 (93.97%)	7.2951 (91.19%)	22.0438 (91.85%)	7.8510 (98.14%)
GAY-6	7.2530 (90.66%)	7.5208 (94.01%)	7.2781 (90.98%)	22.0518 (91.88%)	7.8520 (98.15%)
GAY-mix	7.4489 (93.11%)	7.5739 (94.67%)	7.2972 (91.22%)	22.3200 (93.00%)	7.8611 (98.26%)
					
**Megalomus stangei**					
MEG-1	7.1846 (89.81%)	7.5270 (94.09%)	7.3183 (91.48%)	22.0299 (91.79%)	7.8551 (98.19%)
MEG-2	7.1832 (89.79%)	7.5393 (94.24%)	7.3374 (91.72%)	22.0599 (91.92%)	7.8568 (98.21%)
MEG-3	7.1924 (89.90%)	7.5116 (93.89%)	7.2853 (91.07%)	21.9892 (91.62%)	7.8515 (98.14%)
MEG-mix	7.2022 (90.03%)	7.5468 (94.33%)	7.3570 (91.96%)	22.1059 (92.11%)	7.8574 (98.22%)

## Data Availability

All data are contained within the article.
